# 2,6-Dimethyl­phenyl acridine-9-carboxyl­ate

**DOI:** 10.1107/S160053681205129X

**Published:** 2013-01-04

**Authors:** Damian Trzybiński, Michał Wera, Karol Krzymiński, Jerzy Błażejowski

**Affiliations:** a Faculty of Chemistry, University of Gdańsk, J. Sobieskiego 18, 80-952 Gdańsk, Poland

## Abstract

In the title compound, C_22_H_17_NO_2_, the acridine ring system and the benzene ring are oriented at a dihedral angle of 37.7 (1)°. The carboxyl group is twisted at an angle of 67.7 (1)° relative to the acridine skeleton. In the crystal, mol­ecules are arranged in stacks along the *b* axis, with all of the acridine rings involved in multiple π–π inter­actions [centroid–centroid distances in the range 3.632 (2)–4.101 (2) Å]. The acridine moieties are parallel within the stacks, but inclined at an angle of 52.7 (1)° in adjacent stacks.

## Related literature

For general background, see: Krzymiński *et al.* (2011 ▶); Natrajan *et al.* (2012 ▶). For related structures, see: Sikorski *et al.* (2005 ▶); Sikorski *et al.* (2006 ▶). For inter­molecular inter­actions, see: Hunter *et al.* (2001 ▶). For the synthesis, see: Sato (1996 ▶); Sikorski *et al.* (2005 ▶).
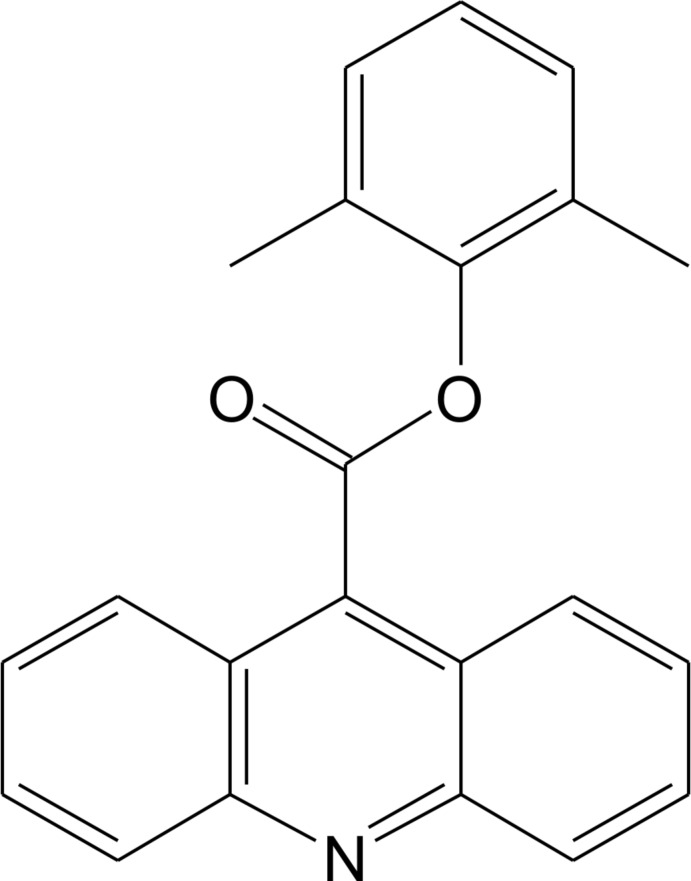


## Experimental

### 

#### Crystal data

C_22_H_17_NO_2_
*M* *_r_* = 327.37
Monoclinic, 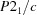

*a* = 12.8617 (10) Å
*b* = 7.5352 (5) Å
*c* = 17.5950 (15) Å
β = 103.143 (8)°
*V* = 1660.6 (2) Å^3^
*Z* = 4
Mo *K*α radiation
μ = 0.08 mm^−1^
*T* = 295 K
0.45 × 0.12 × 0.05 mm

#### Data collection

Oxford Diffraction Gemini R Ultra Ruby CCD diffractometer
Absorption correction: multi-scan (*CrysAlis RED*; Oxford Diffraction, 2008 ▶) *T*_min_ = 0.349, *T*_max_ = 1.000
10387 measured reflections
2913 independent reflections
1908 reflections with *I* > 2σ(*I*)
*R*_int_ = 0.064

#### Refinement

*R*[*F* ^2^ > 2σ(*F* ^2^)] = 0.057
*wR*(*F* ^2^) = 0.151
*S* = 0.97
2913 reflections
228 parameters
H-atom parameters constrained
Δρ_max_ = 0.18 e Å^−3^
Δρ_min_ = −0.24 e Å^−3^

### 

Data collection: *CrysAlis CCD* (Oxford Diffraction, 2008 ▶); cell refinement: *CrysAlis RED* (Oxford Diffraction, 2008 ▶); data reduction: *CrysAlis RED*; program(s) used to solve structure: *SHELXS97* (Sheldrick, 2008 ▶); program(s) used to refine structure: *SHELXL97* (Sheldrick, 2008 ▶); molecular graphics: *ORTEP-3* (Farrugia, 1997 ▶); software used to prepare material for publication: *SHELXL97* and *PLATON* (Spek, 2009 ▶).

## Supplementary Material

Crystal structure: contains datablock(s) global, I. DOI: 10.1107/S160053681205129X/ng5313sup1.cif

Structure factors: contains datablock(s) I. DOI: 10.1107/S160053681205129X/ng5313Isup2.hkl

Supplementary material file. DOI: 10.1107/S160053681205129X/ng5313Isup3.cml

Additional supplementary materials:  crystallographic information; 3D view; checkCIF report

## Figures and Tables

**Fig. 1.**
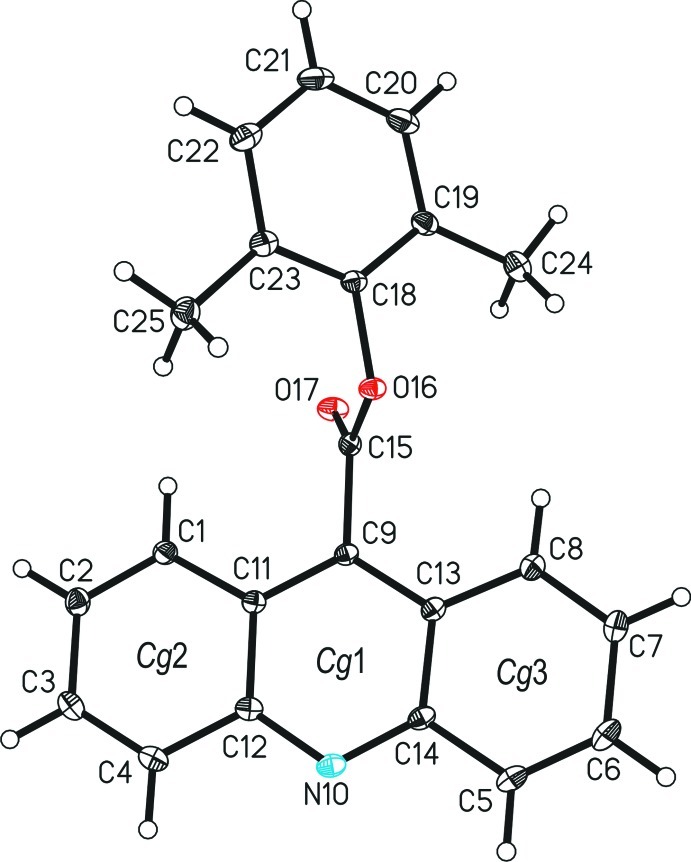
The molecular structure of the title compound showing the atom-labeling scheme. Displacement ellipsoids are drawn at the 25% probability level and H atoms are shown as small spheres of arbitrary radius. Cg1, Cg2 and Cg3 denote the ring centroids.

**Fig. 2.**
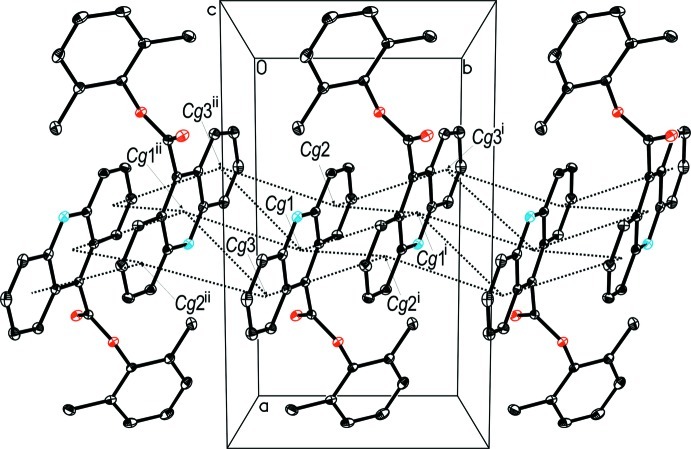
The arrangement of the molecules in the crystal structure. The π–π contacts are represented by dotted lines. H atoms have been omitted. [Symmetry codes: (i) –x + 1, –y + 2, –z; (ii) –x + 1, –y + 1, –z.]

**Fig. 3.**
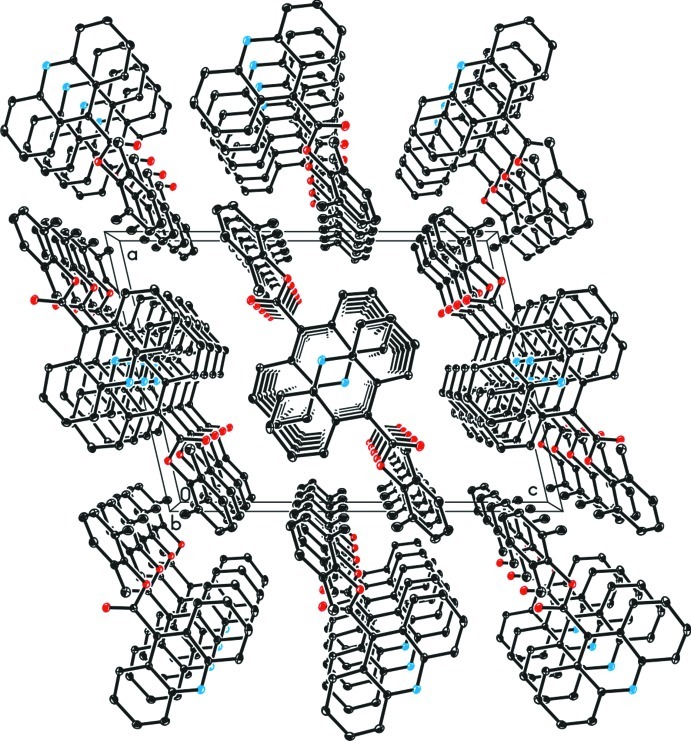
Molecular stacks in the crystal structure, viewed along the b axis. H atoms have been omitted.

## supplementary crystallographic information

### Comment

Phenyl acridine-9-carboxylates are the precursors of 9-(phenoxycarbonyl)-10-methylacridinium salts, whose cations exhibit a chemiluminogenic ability that can be utilized analytically (Natrajan *et al.*, 2012). Here we present the structure of the precursor of one of the chemiluminogens that we have recently investigated (Krzymiński *et al.*, 2011).

The bond lengths and angles characterizing the geometry of the acridine and phenyl moieties of the title compound (Fig. 1) are similar to those found in earlier investigated phenyl acridine-9-carboxylates alkyl-substituted at the benzene ring (Sikorski *et al.*, 2005; Sikorski *et al.*, 2006). With respective average deviations from planarity of 0.0245 (3) Å and 0.0084 (3) Å, the acridine and benzene ring systems are oriented at a dihedral angle of 37.7 (1)° (this angle is equal to 30.0 (2)° in 2-methylphenyl acridine-9-carboxylate (Sikorski *et al.*, 2006) and 35.7 (2)° in 2,5-dimethylphenyl acridine-9-carboxylate (Sikorski *et al.*, 2005)). The carboxyl group is twisted at an angle of 67.7 (1)° relative to the acridine skeleton (this angle is equal to 58.0 (2)° in 2-methylphenyl acridine-9-carboxylate (Sikorski *et al.*, 2006) and 68.1 (2)° in 2,5-dimethylphenyl acridine-9-carboxylate (Sikorski *et al.*, 2005)).

The search for intermolecular interactions in the crystal using PLATON (Spek, 2009) has shown that the inversely related molecules of the title compound (Fig. 2) are arranged in stacks along the *b* axis (Fig. 3) in which all acridine rings are involved in multiple π–π interactions (Table 1, Fig. 2) of an attractive nature (Hunter *et al.*, 2001). The acridine moieties are parallel in stacks but inclined at an angle of 52.7 (1)° in adjacent stacks. The crystal structure is stabilized by dispersive interactions between neighboring stacks. This interesting crystal architecture is unique among the structures of phenyl acridine-9-carboxylates determined to date.

### Experimental

2,6-Dimethylphenyl acridine-9-carboxylate was synthesized by the esterification of 9-(chlorocarbonyl)acridine (obtained in the reaction of acridine-9-carboxylic acid with a tenfold molar excess of thionyl chloride) with 2,6-dimethylphenol in anhydrous dichloromethane in the presence of N,N-diethylethanamine and a catalytic amount of N,N-dimethyl-4-pyridinamine (308–313 K, 25 h) (Sato, 1996; Sikorski *et al.*, 2005). The product was purified chromatographically (SiO_2_, cyclohexane/ethyl acetate, 1/1 v/v). Light-yellow crystals suitable for X-ray investigations were grown from cyclohexane (m.p. 434.5–435.5 K).

### Refinement

H atoms were positioned geometrically, with C–H = 0.93 Å and 0.96 Å for the aromatic and methyl H atoms, respectively, and constrained to ride on their parent atoms with *U*_iso_(H) = *xU*_eq_(C), where *x* = 1.2 for the aromatic and *x* = 1.5 for the methyl H atoms.

### Crystal data

**Table:**

C_22_H_17_NO_2_	*F*(000) = 688
*M**_r_* = 327.37	*D*_x_ = 1.313 Mg m^−^^3^
Monoclinic, *P*2_1_/*c*	Mo *K*α radiation, λ = 0.71073 Å
Hall symbol: -P 2ybc	Cell parameters from 2738 reflections
*a* = 12.8617 (10) Å	θ = 3.5–29.2°
*b* = 7.5352 (5) Å	µ = 0.08 mm^−^^1^
*c* = 17.5950 (15) Å	*T* = 295 K
β = 103.143 (8)°	Needle, light-yellow
*V* = 1660.6 (2) Å^3^	0.45 × 0.12 × 0.05 mm
*Z* = 4	

### Data collection

**Table:**

Oxford Diffraction Gemini R Ultra Ruby CCD diffractometer	2913 independent reflections
Radiation source: Enhanced (Mo) X-ray Source	1908 reflections with *I* > 2σ(*I*)
Graphite monochromator	*R*_int_ = 0.064
Detector resolution: 10.4002 pixels mm^-1^	θ_max_ = 25.0°, θ_min_ = 3.5°
ω scans	*h* = −15→13
Absorption correction: multi-scan (*CrysAlis RED*; Oxford Diffraction, 2008)	*k* = −8→8
*T*_min_ = 0.349, *T*_max_ = 1.000	*l* = −18→20
10387 measured reflections	

### Refinement

**Table:**

Refinement on *F*^2^	Primary atom site location: structure-invariant direct methods
Least-squares matrix: full	Secondary atom site location: difference Fourier map
*R*[*F*^2^ > 2σ(*F*^2^)] = 0.057	Hydrogen site location: inferred from neighbouring sites
*wR*(*F*^2^) = 0.151	H-atom parameters constrained
*S* = 0.97	*w* = 1/[σ^2^(*F*_o_^2^) + (0.0773*P*)^2^] where *P* = (*F*_o_^2^ + 2*F*_c_^2^)/3
2913 reflections	(Δ/σ)_max_ < 0.001
228 parameters	Δρ_max_ = 0.18 e Å^−^^3^
0 restraints	Δρ_min_ = −0.24 e Å^−^^3^

### Special details

**Table:**

Geometry. All esds (except the esd in the dihedral angle between two l.s. planes) are estimated using the full covariance matrix. The cell esds are taken into account individually in the estimation of esds in distances, angles and torsion angles; correlations between esds in cell parameters are only used when they are defined by crystal symmetry. An approximate (isotropic) treatment of cell esds is used for estimating esds involving l.s. planes.

### Fractional atomic coordinates and isotropic or equivalent isotropic displacement parameters (Å^2^)

**Table:**

	*x*	*y*	*z*	*U*_iso_*/*U*_eq_	
C1	0.48802 (17)	0.9058 (3)	0.16670 (14)	0.0453 (6)	
H1	0.4347	0.9142	0.1944	0.054*	
C2	0.58693 (18)	0.9693 (3)	0.19883 (15)	0.0528 (6)	
H2	0.6007	1.0210	0.2481	0.063*	
C3	0.66912 (19)	0.9573 (3)	0.15763 (17)	0.0551 (7)	
H3	0.7365	1.0021	0.1800	0.066*	
C4	0.65119 (18)	0.8819 (3)	0.08637 (16)	0.0516 (6)	
H4	0.7064	0.8749	0.0603	0.062*	
C5	0.4238 (2)	0.5983 (3)	−0.12990 (15)	0.0544 (6)	
H5	0.4820	0.5880	−0.1528	0.065*	
C6	0.3282 (2)	0.5381 (3)	−0.16823 (15)	0.0611 (7)	
H6	0.3209	0.4867	−0.2172	0.073*	
C7	0.2385 (2)	0.5519 (3)	−0.13491 (15)	0.0589 (7)	
H7	0.1724	0.5106	−0.1624	0.071*	
C8	0.24756 (19)	0.6245 (3)	−0.06369 (15)	0.0512 (6)	
H8	0.1878	0.6314	−0.0424	0.061*	
C9	0.36337 (16)	0.7668 (3)	0.05347 (12)	0.0373 (5)	
N10	0.53517 (15)	0.7388 (2)	−0.02047 (12)	0.0463 (5)	
C11	0.46450 (16)	0.8267 (3)	0.09145 (13)	0.0376 (5)	
C12	0.54857 (17)	0.8128 (3)	0.05037 (14)	0.0418 (6)	
C13	0.34688 (17)	0.6905 (3)	−0.02074 (13)	0.0396 (5)	
C14	0.43823 (19)	0.6776 (3)	−0.05490 (13)	0.0428 (6)	
C15	0.27353 (16)	0.7908 (3)	0.09379 (14)	0.0385 (5)	
O16	0.20166 (11)	0.91069 (18)	0.05648 (8)	0.0418 (4)	
O17	0.26699 (12)	0.7193 (2)	0.15308 (10)	0.0545 (5)	
C18	0.12755 (16)	0.9763 (3)	0.09855 (13)	0.0404 (5)	
C19	0.03752 (17)	0.8771 (3)	0.10077 (14)	0.0496 (6)	
C20	−0.03222 (19)	0.9513 (4)	0.14183 (16)	0.0664 (8)	
H20	−0.0937	0.8897	0.1450	0.080*	
C21	−0.0124 (2)	1.1126 (4)	0.17756 (17)	0.0708 (8)	
H21A	−0.0595	1.1581	0.2056	0.085*	
C22	0.0764 (2)	1.2077 (3)	0.17229 (16)	0.0637 (7)	
H22	0.0883	1.3183	0.1961	0.076*	
C23	0.14931 (17)	1.1414 (3)	0.13174 (14)	0.0478 (6)	
C24	0.0152 (2)	0.7020 (3)	0.06043 (18)	0.0698 (8)	
H24A	−0.0550	0.6625	0.0629	0.105*	
H24B	0.0671	0.6166	0.0857	0.105*	
H24C	0.0190	0.7143	0.0068	0.105*	
C25	0.2463 (2)	1.2462 (3)	0.12474 (17)	0.0655 (8)	
H25A	0.2471	1.3573	0.1516	0.098*	
H25B	0.2442	1.2680	0.0706	0.098*	
H25C	0.3096	1.1802	0.1475	0.098*	

### Atomic displacement parameters (Å^2^)

**Table:**

	*U*^11^	*U*^22^	*U*^33^	*U*^12^	*U*^13^	*U*^23^
C1	0.0440 (13)	0.0553 (13)	0.0372 (14)	−0.0005 (11)	0.0104 (11)	−0.0009 (11)
C2	0.0547 (15)	0.0593 (14)	0.0411 (15)	−0.0042 (12)	0.0038 (13)	−0.0002 (12)
C3	0.0416 (13)	0.0585 (15)	0.0618 (19)	−0.0061 (11)	0.0048 (13)	0.0081 (13)
C4	0.0423 (13)	0.0536 (14)	0.0609 (18)	0.0029 (11)	0.0161 (13)	0.0124 (13)
C5	0.0751 (18)	0.0502 (13)	0.0443 (16)	0.0070 (13)	0.0270 (14)	−0.0008 (12)
C6	0.094 (2)	0.0537 (15)	0.0386 (16)	0.0000 (14)	0.0217 (16)	−0.0054 (12)
C7	0.0680 (17)	0.0592 (15)	0.0452 (17)	−0.0065 (12)	0.0039 (14)	−0.0080 (13)
C8	0.0550 (14)	0.0552 (14)	0.0435 (16)	−0.0017 (12)	0.0114 (12)	−0.0056 (12)
C9	0.0416 (13)	0.0384 (11)	0.0332 (13)	0.0032 (9)	0.0113 (10)	0.0033 (10)
N10	0.0515 (12)	0.0487 (11)	0.0426 (13)	0.0089 (9)	0.0189 (10)	0.0079 (9)
C11	0.0399 (12)	0.0414 (11)	0.0313 (13)	0.0025 (10)	0.0079 (10)	0.0058 (10)
C12	0.0441 (13)	0.0428 (12)	0.0401 (15)	0.0065 (10)	0.0130 (11)	0.0117 (11)
C13	0.0471 (13)	0.0381 (11)	0.0340 (13)	0.0042 (10)	0.0100 (11)	0.0035 (10)
C14	0.0548 (14)	0.0415 (12)	0.0346 (14)	0.0086 (11)	0.0154 (11)	0.0057 (10)
C15	0.0395 (12)	0.0421 (12)	0.0336 (14)	0.0000 (10)	0.0073 (10)	0.0014 (10)
O16	0.0417 (8)	0.0498 (8)	0.0358 (9)	0.0086 (7)	0.0126 (7)	0.0059 (7)
O17	0.0548 (10)	0.0681 (10)	0.0449 (11)	0.0129 (8)	0.0204 (8)	0.0177 (9)
C18	0.0384 (12)	0.0497 (13)	0.0331 (13)	0.0104 (10)	0.0083 (10)	0.0045 (10)
C19	0.0388 (13)	0.0628 (15)	0.0465 (16)	0.0048 (11)	0.0078 (11)	0.0052 (12)
C20	0.0425 (15)	0.094 (2)	0.066 (2)	0.0067 (14)	0.0189 (14)	0.0087 (17)
C21	0.0607 (17)	0.096 (2)	0.063 (2)	0.0278 (17)	0.0275 (15)	0.0028 (17)
C22	0.0748 (18)	0.0632 (16)	0.0539 (18)	0.0194 (14)	0.0165 (15)	−0.0042 (13)
C23	0.0513 (14)	0.0520 (14)	0.0402 (15)	0.0098 (11)	0.0104 (12)	0.0049 (11)
C24	0.0542 (16)	0.0745 (17)	0.079 (2)	−0.0130 (14)	0.0115 (15)	−0.0051 (16)
C25	0.0749 (18)	0.0547 (14)	0.0677 (19)	−0.0089 (13)	0.0177 (16)	−0.0063 (14)

### Geometric parameters (Å, º)

**Table:**

C1—C2	1.356 (3)	C11—C12	1.435 (3)
C1—C11	1.420 (3)	C13—C14	1.440 (3)
C1—H1	0.9300	C15—O17	1.194 (2)
C2—C3	1.414 (3)	C15—O16	1.351 (2)
C2—H2	0.9300	O16—C18	1.422 (2)
C3—C4	1.348 (3)	C18—C23	1.376 (3)
C3—H3	0.9300	C18—C19	1.386 (3)
C4—C12	1.426 (3)	C19—C20	1.391 (3)
C4—H4	0.9300	C19—C24	1.495 (3)
C5—C6	1.340 (3)	C20—C21	1.366 (4)
C5—C14	1.422 (3)	C20—H20	0.9300
C5—H5	0.9300	C21—C22	1.370 (4)
C6—C7	1.412 (4)	C21—H21A	0.9300
C6—H6	0.9300	C22—C23	1.394 (3)
C7—C8	1.348 (3)	C22—H22	0.9300
C7—H7	0.9300	C23—C25	1.505 (3)
C8—C13	1.418 (3)	C24—H24A	0.9600
C8—H8	0.9300	C24—H24B	0.9600
C9—C11	1.395 (3)	C24—H24C	0.9600
C9—C13	1.398 (3)	C25—H25A	0.9600
C9—C15	1.498 (3)	C25—H25B	0.9600
N10—C14	1.338 (3)	C25—H25C	0.9600
N10—C12	1.340 (3)		
			
C2—C1—C11	121.2 (2)	N10—C14—C5	118.4 (2)
C2—C1—H1	119.4	N10—C14—C13	123.6 (2)
C11—C1—H1	119.4	C5—C14—C13	118.0 (2)
C1—C2—C3	120.2 (2)	O17—C15—O16	123.36 (19)
C1—C2—H2	119.9	O17—C15—C9	125.1 (2)
C3—C2—H2	119.9	O16—C15—C9	111.52 (18)
C4—C3—C2	120.9 (2)	C15—O16—C18	116.42 (16)
C4—C3—H3	119.5	C23—C18—C19	124.5 (2)
C2—C3—H3	119.5	C23—C18—O16	116.07 (19)
C3—C4—C12	120.8 (2)	C19—C18—O16	119.39 (19)
C3—C4—H4	119.6	C18—C19—C20	116.1 (2)
C12—C4—H4	119.6	C18—C19—C24	122.3 (2)
C6—C5—C14	121.3 (2)	C20—C19—C24	121.6 (2)
C6—C5—H5	119.3	C21—C20—C19	121.5 (2)
C14—C5—H5	119.3	C21—C20—H20	119.3
C5—C6—C7	120.6 (2)	C19—C20—H20	119.3
C5—C6—H6	119.7	C20—C21—C22	120.4 (2)
C7—C6—H6	119.7	C20—C21—H21A	119.8
C8—C7—C6	120.6 (2)	C22—C21—H21A	119.8
C8—C7—H7	119.7	C21—C22—C23	121.1 (2)
C6—C7—H7	119.7	C21—C22—H22	119.5
C7—C8—C13	121.0 (2)	C23—C22—H22	119.5
C7—C8—H8	119.5	C18—C23—C22	116.5 (2)
C13—C8—H8	119.5	C18—C23—C25	122.1 (2)
C11—C9—C13	120.48 (19)	C22—C23—C25	121.4 (2)
C11—C9—C15	118.01 (19)	C19—C24—H24A	109.5
C13—C9—C15	121.49 (19)	C19—C24—H24B	109.5
C14—N10—C12	118.28 (18)	H24A—C24—H24B	109.5
C9—C11—C1	124.1 (2)	C19—C24—H24C	109.5
C9—C11—C12	117.5 (2)	H24A—C24—H24C	109.5
C1—C11—C12	118.37 (19)	H24B—C24—H24C	109.5
N10—C12—C4	118.4 (2)	C23—C25—H25A	109.5
N10—C12—C11	123.1 (2)	C23—C25—H25B	109.5
C4—C12—C11	118.5 (2)	H25A—C25—H25B	109.5
C9—C13—C8	124.7 (2)	C23—C25—H25C	109.5
C9—C13—C14	116.9 (2)	H25A—C25—H25C	109.5
C8—C13—C14	118.4 (2)	H25B—C25—H25C	109.5
			
C11—C1—C2—C3	−0.2 (3)	C6—C5—C14—N10	−178.5 (2)
C1—C2—C3—C4	−0.5 (3)	C6—C5—C14—C13	0.4 (3)
C2—C3—C4—C12	0.3 (3)	C9—C13—C14—N10	−2.0 (3)
C14—C5—C6—C7	0.0 (4)	C8—C13—C14—N10	178.56 (19)
C5—C6—C7—C8	−0.6 (4)	C9—C13—C14—C5	179.15 (18)
C6—C7—C8—C13	0.7 (4)	C8—C13—C14—C5	−0.3 (3)
C13—C9—C11—C1	179.93 (19)	C11—C9—C15—O17	−65.5 (3)
C15—C9—C11—C1	1.3 (3)	C13—C9—C15—O17	115.9 (2)
C13—C9—C11—C12	2.3 (3)	C11—C9—C15—O16	111.8 (2)
C15—C9—C11—C12	−176.36 (18)	C13—C9—C15—O16	−66.8 (2)
C2—C1—C11—C9	−176.7 (2)	O17—C15—O16—C18	12.0 (3)
C2—C1—C11—C12	1.0 (3)	C9—C15—O16—C18	−165.32 (17)
C14—N10—C12—C4	−178.49 (18)	C15—O16—C18—C23	100.9 (2)
C14—N10—C12—C11	1.1 (3)	C15—O16—C18—C19	−82.0 (2)
C3—C4—C12—N10	−179.8 (2)	C23—C18—C19—C20	−2.0 (3)
C3—C4—C12—C11	0.6 (3)	O16—C18—C19—C20	−178.9 (2)
C9—C11—C12—N10	−2.9 (3)	C23—C18—C19—C24	177.0 (2)
C1—C11—C12—N10	179.25 (18)	O16—C18—C19—C24	0.1 (3)
C9—C11—C12—C4	176.66 (18)	C18—C19—C20—C21	0.1 (4)
C1—C11—C12—C4	−1.2 (3)	C24—C19—C20—C21	−178.9 (3)
C11—C9—C13—C8	179.43 (19)	C19—C20—C21—C22	1.4 (4)
C15—C9—C13—C8	−2.0 (3)	C20—C21—C22—C23	−1.1 (4)
C11—C9—C13—C14	0.0 (3)	C19—C18—C23—C22	2.3 (3)
C15—C9—C13—C14	178.56 (18)	O16—C18—C23—C22	179.24 (19)
C7—C8—C13—C9	−179.7 (2)	C19—C18—C23—C25	−177.5 (2)
C7—C8—C13—C14	−0.2 (3)	O16—C18—C23—C25	−0.6 (3)
C12—N10—C14—C5	−179.71 (18)	C21—C22—C23—C18	−0.7 (4)
C12—N10—C14—C13	1.4 (3)	C21—C22—C23—C25	179.2 (2)

### π–π interactions (Å, °).

*Cg*1, *Cg*2 and *Cg*3 are the centroids of the C9/N10/C11–C14, C1–C4/C11/C12 and C5–C8/C13/C14 rings, respectively. Cg*I*···Cg*J* is the distance between ring centroids. The dihedral angle is that between the planes of the rings *I* and *J*. *CgI*_Perp is the perpendicular distance of *CgI* from ring *J*. Cg*I*_Offset is the distance between *CgI* and perpendicular projection of *CgJ* on ring *I*.

**Table:**

*I*	*J*	*CgI*···*CgJ*	Dihedral angle	*CgI*_Perp	Cg*I*_Offset
1	1^i^	4.040 (2)	0.0 (2)	3.396 (1)	2.188 (2)
1	1^ii^	4.101 (2)	0.0 (2)	3.375 (1)	2.330 (2)
1	2^i^	3.632 (2)	2.0 (2)	3.348 (1)	1.408 (2)
1	3^ii^	3.914 (2)	1.1 (2)	3.338 (1)	2.044 (2)
2	1^i^	3.632 (2)	2.0 (2)	3.373 (1)	1.347 (2)
2	3^i^	3.990 (2)	3.0 (2)	3.400 (1)	2.088 (2)
2	3^ii^	4.071 (2)	3.0 (2)	3.365 (1)	2.291 (2)
3	1^ii^	3.914 (2)	1.1 (2)	3.371 (1)	1.989 (2)
3	2^i^	3.990 (2)	3.0 (2)	3.306 (1)	2.234 (2)
3	2^ii^	4.071 (2)	3.0 (2)	3.413 (1)	2.219 (2)

Symmetry codes: (i) –*x* + 1, –*y* + 2, –*z*; (ii) –*x* + 1, –*y* + 1, –*z*.
